# Supplementary material for: ZO-1 Regulates Hippo-Independent YAP Activity and Cell Proliferation via a GEF-H1- and TBK1-Regulated Signalling Network
Source: Cells. 2024 Apr 5;13(7):640. doi: 10.3390/cells13070640 (PMC11011562; doi:10.3390/cells13070640)
Supplement: Supplementary file 1 [file cells-13-00640-s001.zip › cells-2908453-supplementary.pdf]

Supplementary Figures

ZO-1 regulates Hippo-independent YAP activity and cell proliferation via a GEF-H1- and TBK1-regulated signalling network

Alexis J. Haas, Mert Karakus, Ceniz Zihni, Maria S. Balda and Karl Matter

*** Correspondence:** Karl Matter: k.matter@ucl.ac.uk / Maria S. Balda: m.balda@ucl.ac.uk

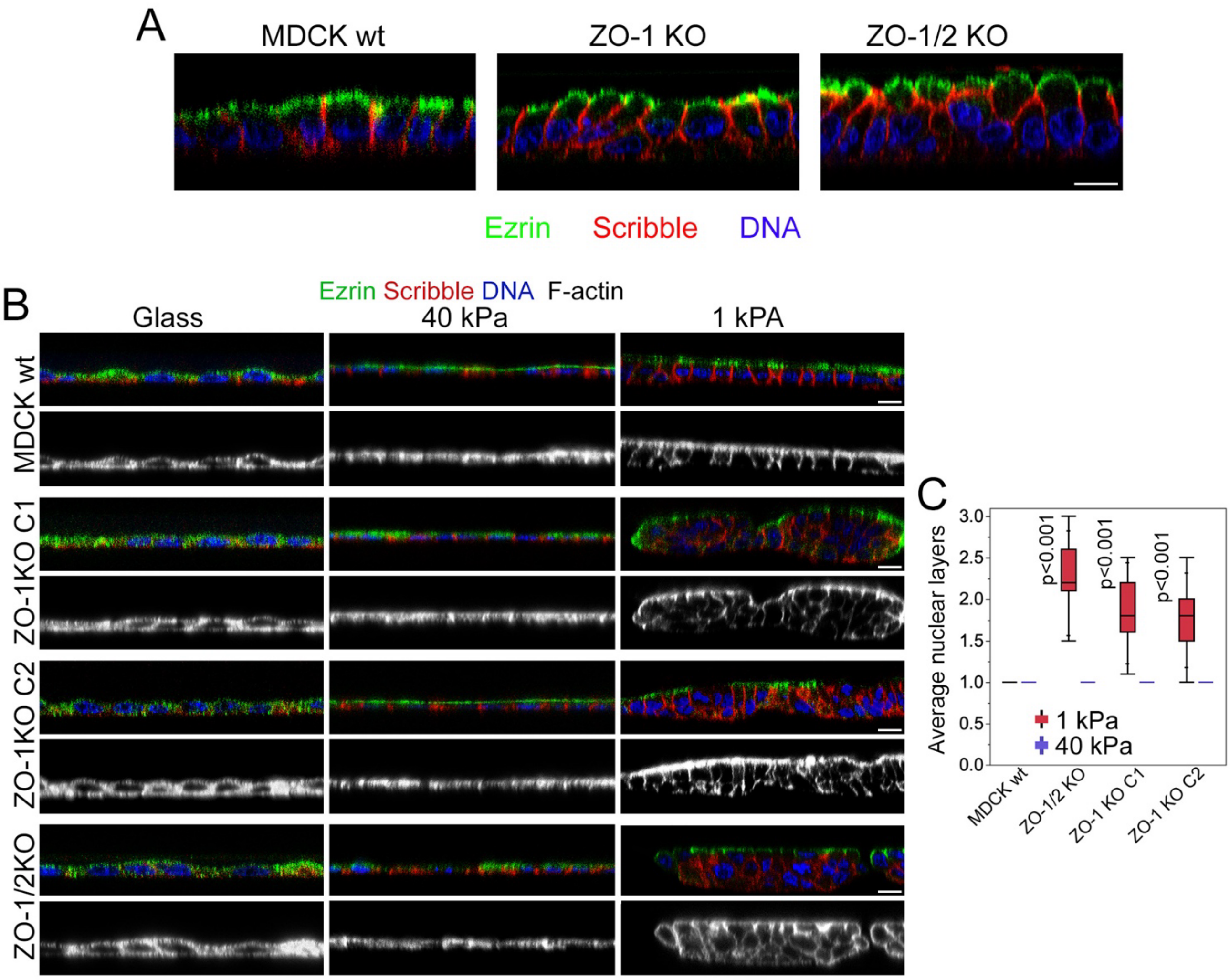

Figure S1. Knockout of ZO-1 increases cell proliferation and disrupts monolayer organization.

Expression of polarity markers and monolayer organization by control and knockout MDCK cells grown on filters (**A**) or hydrogels (**B**,**C**). Quantification shows means, interquartile ranges, and p-values derived from a signed-rank test (n=15 images per cell line and condition). Magnification bars, 20 μm.

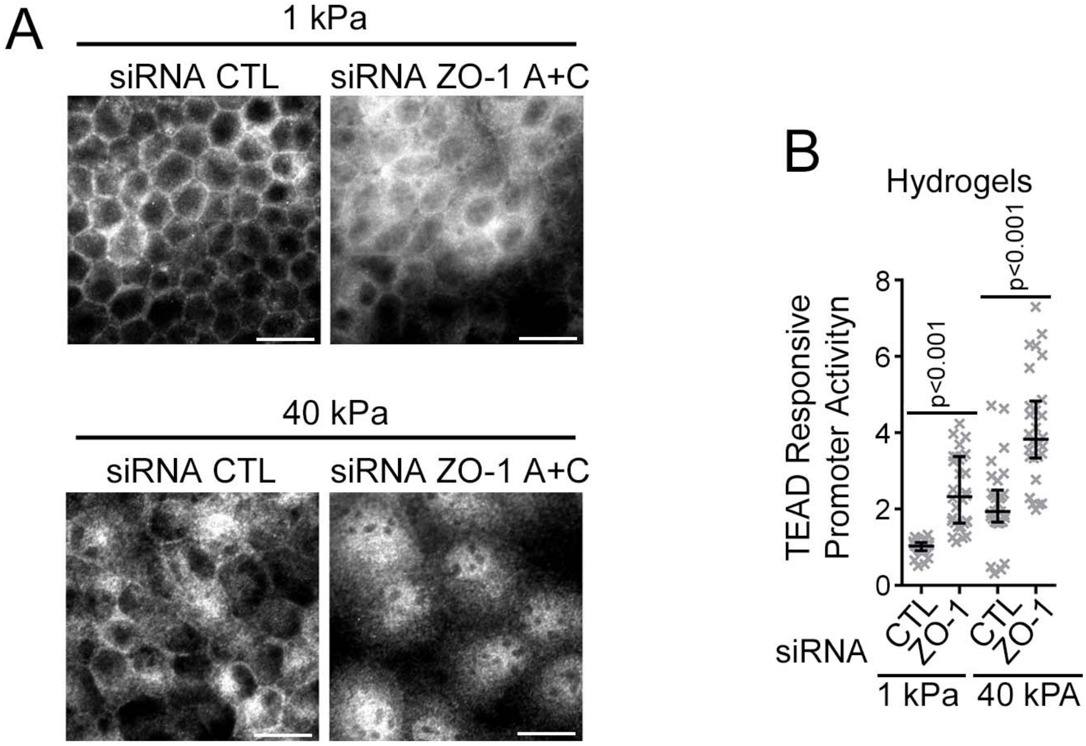

Figure S2. Knockdown of ZO-1 promotes nuclear translocation of YAP.

A siRNA-transfected MDCK cells grown on 40 and 1 kPa PAA hydrogels were fixed and immunostained for YAP. **B** TEAD transcriptional activity was analyzed by reporter gene assay in MDCK cells grown on hydrogels of 40 and 1 kPa. The quantification shows individual cells analyzed, medians, interquartile ranges, and p-values derived from Wilcoxon tests. Magnification bars, 20 μm.

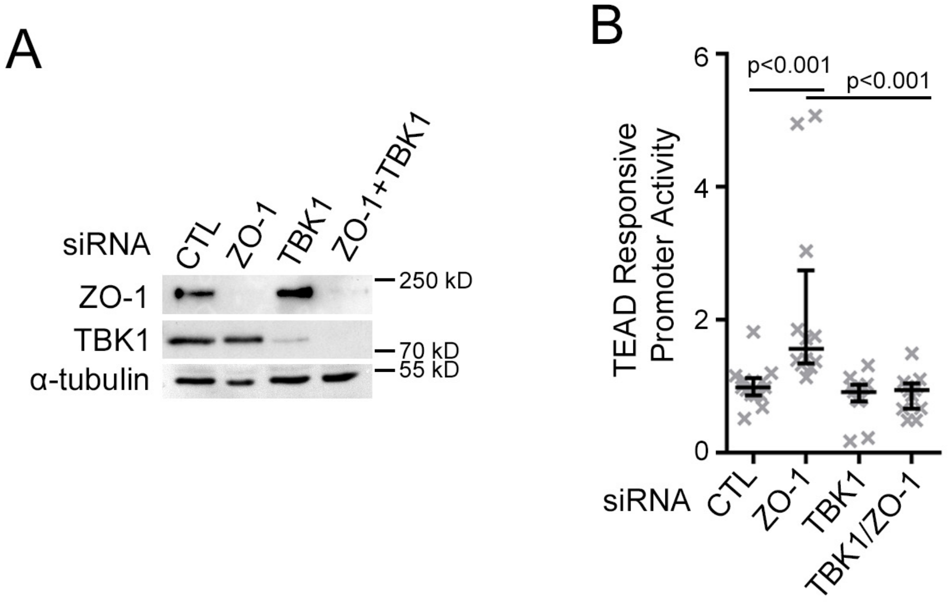

Figure S3. Knockdown of TBK1 inhibits YAP/TEAD activation in ZO-1 depleted cells.

MDCK cells were transfected with siRNAs as indicated and then analyzed by immunoblotting (**A**) or a TEAD responsive promoter assay (**B**). The reporter assay shows individual determinations, medians, interquartile ranges, and p-values for the indicated pairs from Wilcoxon tests.

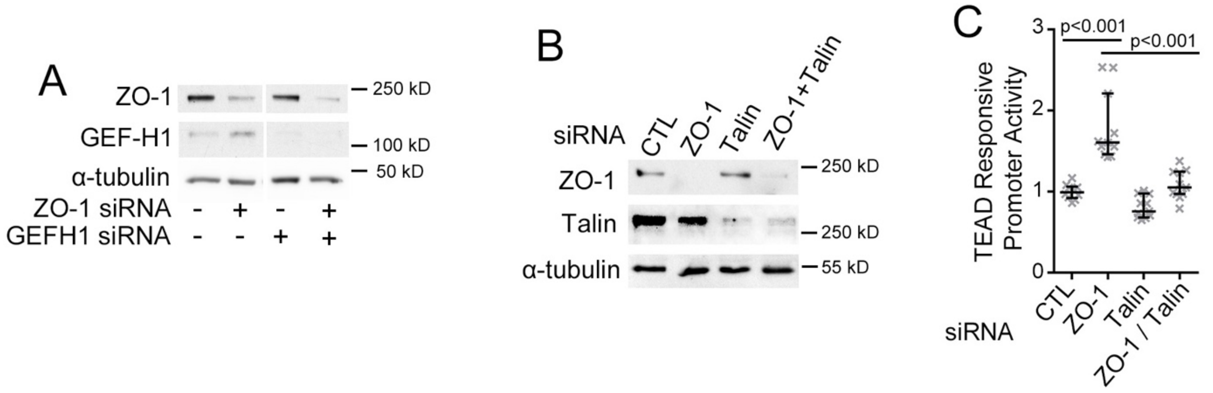

Figure S4. Analysis of siRNA transfected MDCK cells.

MDCK cells were transfected with siRNAs as indicated and then analyzed by immunoblotting to determine GEF-H1 and ZO-1 expression (**A**), knockdown of ZO-1 and talin (**B**), or the impact on the TEAD responsive promoter gene assay (**C**, shown are individual determinations, medians, interquartile ranges and p-values derived from Wilcoxon tests).
